# Integrins in cancer stem cells

**DOI:** 10.3389/fcell.2024.1434378

**Published:** 2024-08-21

**Authors:** Siqi Gou, Anqi Wu, Zhigang Luo

**Affiliations:** ^1^ The Second Affiliated Hospital, Department of urology, Hengyang Medical School, University of South China, Hengyang, China; ^2^ The Second Affiliated Hospital, Department of Clinical Research Center, Hengyang Medical School, University of South China, Hengyang, China

**Keywords:** integrin, cancer, stem cell, stemness, stemness marker

## Abstract

Integrins are a class of adhesion receptors on cell membranes, consisting of α and β subunits. By binding to the extracellular matrix, integrins activate intracellular signaling pathways, participating in every step of cancer initiation and progression. Tumor stem cells possess self-renewal and self-differentiation abilities, along with strong tumorigenic potential. In this review, we discussed the role of integrins in cancer, with a focus on their impact on tumor stem cells and tumor stemness. This will aid in targeting tumor stem cells as a therapeutic approach, leading to the exploration of novel cancer treatment strategies.

## 1 Introduction

Cell-extracellular matrix (ECM) interactions are crucial processes for cell growth, differentiation, and function, regulating cell behavior and characteristics through various signaling pathways, playing indispensable roles in developmental morphogenesis and physiological functions (Humphrey, Dufresne, and Schwartz, 2014; Le Saux et al., 2020). Moreover, ECM is an essential component of the stem cell niche (Chen, Lewallen, and Xie, 2013). Cell-cell and cell-ECM adhesion within the stem cell niche are not only crucial for establishing and maintaining niche structure, but also for generating and transmitting short-range regulatory signals, and controlling the frequency and nature of stem cell division. In many stem cell niches, adhesion to supporting cells and/or the ECM determines the orientation of stem cell division planes, thereby aiding in the regulation of stem cell self-renewal and differentiation (Raymond et al., 2009). Stem cells can only maintain their capacity for self-renewal and proliferation within their microenvironment, where cell adhesion constitutes a central aspect of cell-ECM interactions. Thus, cell adhesion molecules such as cadherins, integrins, among others, have become crucial mediators anchoring stem cells within their microenvironment (Chen, Lewallen, and Xie, 2013). In addition, stem cell adhesion and its downstream signaling pathways have become crucial indicators for tissue regeneration and repair (Maynard et al., 2021; Mariadoss and Wang, 2023).

According to their structural characteristics, cell adhesion molecules can be classified into several major families, including the integrin family, cadherin family, selectin family, immunoglobulin superfamily, mucin-like vascular addressins, as well as some unclassified molecules such as peripheral lymph node addressin (PNAd), cutaneous lymphocyte antigen (CLA), and CD44, among others. In mediating interactions within the stem cell niche, the cell adhesion molecules primarily involved are from the cadherin and integrin families (Chen, Lewallen, and Xie, 2013).

Collagen is a primary component of the ECM, providing structural and mechanical support to cells (Somaiah et al., 2015; He et al., 2018; Chung et al., 2024). In addition, collagen is closely associated with cell adhesion, migration, differentiation, signal transduction, and maintenance of the microenvironment. In stem cells, collagen also plays a role in maintaining the stem cell niche by regulating interactions between stem cells and the surrounding ECM. Integrins and discoidin domain receptors (DDRs) are the two most important types of receptors for collagen (Yeh, Lin, and Tang, 2012), facilitating interactions between cells and collagen. Integrins were the first collagen receptors discovered. Integrins α1β1, α2β1, α3β1, α10β1, and α11β1 are all capable of binding collagen and play roles in cell adhesion (Yeh, Lin, and Tang, 2012; Hynes, 2002; Takada et al., 2007). DDR1 and DDR2, identified subsequently as another class of collagen receptors (Agarwal, Smith, and Jones, 2019; W.F; Vogel, Abdulhussein, and Ford, 2006), play significant roles in various aspects. DDR1, also known as cell adhesion kinase 1 (CAK1), activates integrin-mediated cell adhesion enhancement (Xu et al., 2012; W; Vogel et al., 2000) and can also promote cell adhesion in an integrin-independent manner (Dejmek et al., 2003). DDR1 binds collagen to stimulate ECM, regulate osteoblast activity, and promote bone remodeling and regeneration. Moreover, through various mechanisms including regulating tumor cell adhesion, migration, signal transduction, and cancer stem cell characteristics, DDR1 plays a crucial role in the development and progression of tumors (Y.X. Xiong et al., 2023; Curat and Vogel, 2002; Heinzelmann-Schwarz et al., 2004). DDR2 primarily participates in collagen synthesis and turnover, contributing to tissue regeneration and repair (Kim et al., 2013; Mariadoss and Wang, 2023). DDRs and integrins have been extensively studied in cancer and are currently important targets for cancer therapy (Bibi et al., 2024; Gupta et al., 2022). Next, we will primarily discuss the ECM receptor - integrins, including their roles in tumor initiation, progression, and within cancer stem cells.

**FIGURE 1:**
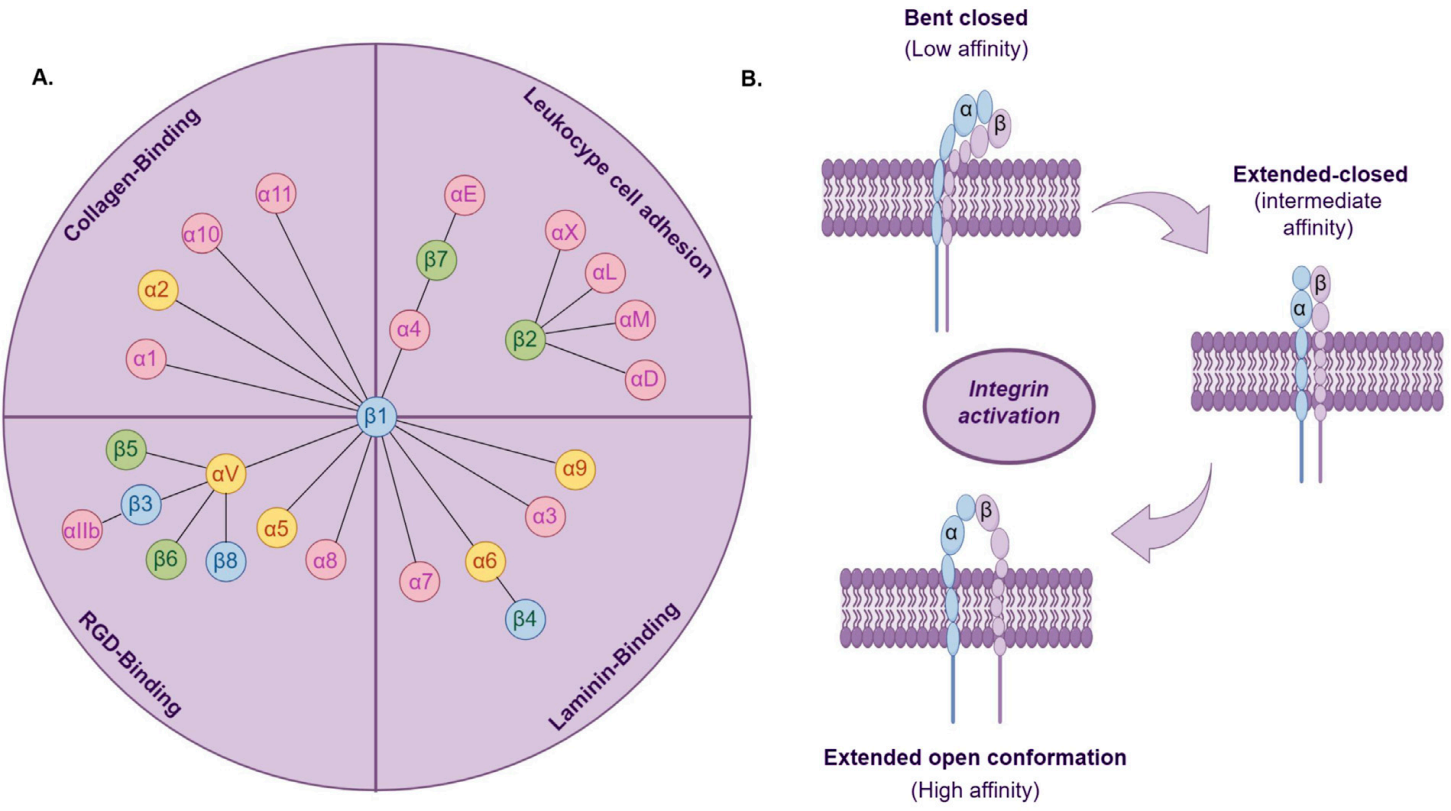
**(A)** The 24 integrin heterodimers are classified based on combinations of α and β subunits, specific ligands, or cell types. Yellow marks indicate integrin a subunits associated with cancer stem cells; blue marks indicate integrin β subunits associated with cancer stem cells. **(B)**. Three conformational states of integrin activation.

A large extracellular domain (ectodomain), a transmembrane domain (TM), and a typical small cytoplasmic tail (CT) collectively constitute an integrin subunit. As a class of transmembrane proteins, the integrin family of 24 transmembrane heterodimers consisting of non-covalent linkages of the α- and β-subunits (ITGA and ITGB) (Figure 1A) exists to mediate cellular interactions with the ECM by concurrently binding to extracellular ECM proteins and establishing a linkage to the intracellular actin cytoskeleton (Hynes, 2002). In many biological functions mediated by integrins, regulating their adhesion strength to the extracellular matrix (ECM) is crucial. The adhesion strength mediated by integrins can be regulated through two main mechanisms: clustering of integrins and conformational changes in integrins, known as integrin activation. In addition to mediating cell adhesion, integrins can also facilitate bidirectional signaling through binding to extracellular ligands or interacting with the cell cytoskeleton via their intracellular domains. This dual signaling capability from outside to inside and inside to outside is a distinctive feature of integrins (Bökel and Brown, 2002).

Integrin activation is the transition from a low-affinity to a high-affinity ligand-binding state (Figure 1B). Studies indicate that integrins typically exist in a low-affinity state for ligand binding. Only when stimulated by internal or external signals do they undergo conformational changes to a higher-affinity state for ligand binding (Cooper and Giancotti, 2019). Based on their binding characteristics, integrins can be classified into four main types: leukocyte cell adhesion integrins, RGD-binding integrins, collagen-binding integrins, and laminin-binding integrins (Humphries, Byron, and Humphries, 2006). The extracellular domains of integrins bind to extracellular ligands, recruiting adaptor proteins, cytoskeletal proteins, and signaling molecules to the cytoplasmic tail, thereby initiating external signal transduction (Pang et al., 2023). Integrins are expressed in all multicellular organisms. During evolution, the expression of integrins exhibits diversity and specificity, establishing a crucial foundation for their role in biological evolution. The evolution of vertebrate integrin subunits has arisen from the evolution of homologous sequences and structural domains. These sequences and domains originally functioned in cell adhesion in early deuterostomes and are also present in protists and prokaryotes (Johnson et al., 2009). Moreover, in single-celled organisms, the presence of integrin heterodimers, ITGA, and ITGB has also been identified (Kang et al., 2021). Many species of amoebas possess either ITGA or ITGB individually, yet they possess all signaling components of integrin-mediated adhesion complexes (IMAC). These amoebas with single integrins may initiate integrin signaling pathways through similar processes and similarly exhibit adhesive functions (Kang et al., 2021). Furthermore, integrins also mediate morphogenesis and organ formation, and their presence has been detected in various organs, embryonic development, and nervous system development (De Arcangelis and Georges-Labouesse, 2000).

Importantly, the occurrence of diseases is equally closely associated with integrin dysregulation. Integrins are widely expressed in the body and mediate various life activities. For example, KOIVISTO et al. found that integrin αvβ6 is expressed in epithelial cells and is associated with the maintenance of intestinal barrier, lung inflammation response, hair follicle development, maintenance of hair follicle stem cell quiescence, and retention of epidermal immune cells (Koivisto et al., 2018). PRIMO et al. discovered that integrin α6 is expressed in endothelial cells and promotes the generation of blood vessels on the basement membrane gel (Primo et al., 2010). Additionally, as membrane proteins, activated integrins play important roles in various chemical and mechanical signal transductions, actively influencing cell proliferation, differentiation, and apoptosis. Therefore, dysregulation of integrins can lead to various diseases. Cancer is a disease influenced by multiple factors, characterized by high metastasis, invasion, recurrence, and resistance to treatment. The interaction between tumor stem cells and the extracellular matrix (ECM) plays a crucial role in the malignant features of cancer, promoting tumor development, invasion, and drug resistance by influencing the biological behavior and signaling pathways of tumor stem cells. In this review, we extensively discuss the significant role of integrins in tumor stemness. We elaborate on integrins as markers for tumor stemness, their capability to isolate and enrich tumor stem cells, and their role as regulatory factors in maintaining the Cancer stem cell phenotype.

## 2 Integrin activator and inhibitor

Talin and Kindlin are key intracellular activators of integrins. Talin regulates integrin affinity and provides mechanical linkage between integrins and the actin cytoskeleton (Hynes, 2002). Talin activation enables the binding of talin-H (N-terminal FERM domain) to integrin-β CT, disrupting integrin-α/β CT binding and triggering receptor conformational activation (Lu et al., 2022). Kindlin also participates in integrin activation by enhancing talin’s interaction with the membrane proximal site through binding to the distal end site of the β integrin cytoplasmic tail, leading to dissociation of α and β integrin cytoplasmic tails (Haydari et al., 2020). The integrin-binding site on Talin-H is masked by the C-terminal rod domain of Talin-R, which, when dimerized, activates Talin and facilitates its connection with integrin co-activator Kindlin-2 via adaptor protein paxillin, promoting integrin clustering and enhancing integrin activation more effectively (Lu et al., 2022). In addition to these, other integrin activators include RGD peptides and analogs, specific monoclonal antibodies, and biologically active proteins (Hynes, 2002; Ruoslahti and Pierschbacher, 1987; Shattil, Kim, and Ginsberg, 2010; Ginsberg, 2014).

Integrin inhibitors are a class of compounds or proteins that can suppress integrin function or its binding with ligands. The primary target of integrin inhibitors is cellular adhesion functions. Known integrin inhibitors include RGD peptide antagonists, small molecule compounds, certain integrin antibodies, and others. Particularly challenging is the RGD-binding subfamily of αv integrins (Desgrosellier and Cheresh, 2010; Slack et al., 2022).

## 3 The role of integrins in tumor progression and targeted integrin therapies

### 3.1 Integrins in tumor development

The abnormal expressed integrins take part in multistep of cancer progression, including initiation, proliferation, angiogenesis, invasion, survival in circulation system, formation of metastatic niche, colonization, immunosuppression, and resistance of treatment.

For instance, asymmetric division with growth initiation is a fundamental feature, with ITGA6 localized specifically on one side of the cell. Previous studies have shown that the polarized distribution of ITGA6 during asymmetric division strongly influences growth initiation (Morris et al., 2020). In metastatic colorectal cancer, the integrin α2β1 signal interacts with Cadherin-17 (CDH17), promoting activation of ITGB1 and recruitment of Talin, thereby facilitating proliferation and metastasis of metastatic colorectal cancer (Bartolomé et al., 2014). In triple-negative breast cancer (TNBC), research has identified interactions between ITGB4, TNFAIP2, and IQGAP1. Through TNFAIP2 and IQGAP1, activation of RAC1 is promoted, which enhances TNBC resistance to therapy and DNA damage repair (H. Fang et al., 2023). Small extracellular vesicles (sEVs) are considered important mediators of angiogenesis (Sato et al., 2019). sEVs released by prostate cancer cells express epithelial-specific integrin αvβ6. αvβ6-positive sEVs can modulate angiogenesis signals in microvascular endothelial cells, promoting angiogenesis in prostate cancer (Krishn et al., 2020). ITGB3 is crucial for tumor invasion, neovascularization, and inflammation. In a mouse model lacking ITGB3, studies have found increased M2 macrophages promoting tumor growth, decreased CD8^+^ T cells, and weakened immune responses. Additionally, ITGB3 signaling favors M1 polarization via STAT1 signaling while inhibiting M2 polarization via STAT6 signaling. Loss of ITGB3 signaling promotes immune-suppressive tumor microenvironments by enhancing M2 TAM polarization and function and reducing the number of CD8^+^ T cells (Krishn et al., 2020).

Tumor metastasis involves five processes: local invasion, intravasation, survival in the circulation, extravasation, and micrometastasis formation (Labelle and Hynes, 2012). Integrins are involved in almost every step of the metastatic cascade. Tumor cells breach the basement membrane and extracellular matrix (ECM) at the primary site with the cooperative action of calcium-binding proteins and cadherins, entering surrounding blood vessels to initiate metastasis (Micalizzi, Maheswaran, and Haber, 2017). ECM serves as the primary ligand recognized by integrins, mediating ECM remodeling through activation of downstream pathways, thereby influencing tumor metastasisIntegrin Signaling in Glioma Pathogenesis: From Biology to Ther(Micalizzi, Maheswaran, and Haber, 2017). Upon transportation via bloodstream to distant tissues, tumor cells establish residence through extravasation and a series of adhesion processes in the distant tissue. For instance, induction of the c-Met/β1 complex promotes intravasation and vascular wall adhesion of triple-negative breast cancer cells (Lau et al., 2021). Blocking integrin α2β1 in cell vesicles can affect the formation of premetastatic niches in the lungs (Kong et al., 2019), while ITGA5 has been shown to interact with RUNX2 to promote bone colonization of breast cancer cells (X.Q. Li et al., 2016).

### 3.2 Regulation mechanisms of integrins

Most studies suggest that integrins primarily regulate and maintain tumor characteristics such as proliferation, migration, and invasion by activating various signaling pathways including TGF/β, FAK-AKT, or ERK (Kemper et al., 2021; Haas et al., 2017; McClure et al., 2019; Gharibi et al., 2017; Lafauy et al., 2021; Korovina et al., 2022). They can also rely on ligand binding to modulate tumor cell proliferation, migration, and invasion by altering integrin conformation and function. Unlike other studies, integrin α2β1 acts as a regulator of p38 MARK kinase phosphorylation, promoting both cell proliferation and inhibition in the process of regulating tumor proliferation. OJALILL et al. found that the proliferation rate of the α2β1high subgroup in prostate cancer cells was slower than that of the α2β1low subgroup, but α2β1 could promote their survival, invasion, and drug resistance (Ojalill et al., 2018).

Epithelial-mesenchymal transition (EMT) is the first step in cancer metastasis and invasion, and one of the key programs for tumor recurrence. Through the EMT process, epithelial cells lose cell-cell connections, weaken apical-basal polarity, and gain the ability to migrate and invade the basement membrane and blood vessels (Jolly et al., 2015; Christiansen and Rajasekaran, 2006). Studies have shown that integrins can bind to integrin-linked kinase to induce AKT activation, thereby inhibiting GSK-3β(Cordes, 2004). GSK-3β is one of the factors influencing tumor stemness (C. Wang et al., 2023).

In addition, integrins also participate in tumor microenvironment formation through a series of processes involving vascular and lymphatic genesis, connective tissue proliferation, and inflammation-related cells.

Integrins are commonly used cell surface markers to distinguish different stem and progenitor cell populations during fluorescence-activated cell sorting. For instance, CD49f (ITGA6) has been successfully used to isolate stem cells in skin and breast tissues (Anderson, Owens, and Naylor, 2014), while ITGB1 has been utilized as a marker for enriching epithelial populations of breast stem cells (Isomursu et al., 2019). Additionally, integrins are believed to interact with the extracellular matrix (ECM) of stem cell niches, mediating mechanical signal transduction and balancing self-renewal and differentiation of stem cells.

### 3.3 Targeting integrins for cancer therapy

Integrins can be targeted through various mechanisms aimed at activating integrin complexes as agonists or deactivating them as antagonists or inhibitors, thereby inhibiting secondary biological processes initiated by integrins (Slack et al., 2022). Additionally, integrins can serve as drug conjugates to deliver cytotoxic drugs in a cell-specific manner, or can be directly incorporated into antigen receptor T cells (CAR T cells) for immunotherapy. While the fundamental principles of targeting integrins for cancer therapy are widely recognized, significant challenges persist in clinical trials (Slack et al., 2022). Currently, integrins targeted for cancer therapy include αv integrins, α5β1, α2, αLβ2, α4β1, α3β1, and β7 (Pang et al., 2023). Table 1 illustrates the mechanisms and targets of integrin targeting in cancer therapy.

**TABLE 1:** Integrins can be targeted against cancers.

Target	Mode of action	Tumor	References
αVβ3	Inhibiting the role of αvβ3 in tumor angiogenesis Inhibiting the upregulation of αvβ3 in endothelial cells; Inhibiting the expression of PDL1 in the tumor microenvironment	Breast cancer Glioblastoma	Stupp et al., 2014; Jin and Varner, 2004; Brown and Marshall, 2019
αVβ5	Inhibiting the role of αvβ3 in tumor angiogenesis Inhibiting the upregulation of αvβ3 in endothelial cells	Breast cancer Glioblastoma	Stupp et al., 2014; Jin and Varner, 2004
αVβ6	Modulating or inhibiting the transforming growth factor-beta (TGF-β) pathway Inducing T cell-mediated immunity	Colorectal cancer Melanoma Pancreatic cancer Breast cancer	Élez et al., 2015; O'Day et al., 2011; Reader et al., 2019; Moore et al., 2020; Bagati et al., 2021; Dong et al., 2017
αVβ8	Inhibiting the activation of TGF-β Enhancing cytotoxic T cell responses in tumors	Squamous cell carcinoma Breast cancer Colon cancer Prostate cancer	Dodagatta-Marri et al. (2021)
αLβ2	Inducing conformational activation of integrins to enhance T cell infiltration into tumors	Melanoma Colorectal cancer	Hailemichael et al. (2019)
α4β1	Inducing conformational activation of integrins to enhance T cell infiltration into tumors	Melanoma Colorectal cancer	Hailemichael et al. (2019)
αIIbβ3	Preventing RGD protein binding to αIIbβ3 to inhibit platelet aggregation Blocking αIIbβ3 activation to indirectly inhibit α2β1 binding to collagen	Prostate cancer Kidney cancer	Bartolomé et al., 2021; L. Zhang, Shan, et al., 2019
α5β1	Blocking the interaction between α5β1 and fibronectin Inhibiting tumor angiogenesis	Kidney cancer Pancreatic cancer Malignant melanoma Lung cancer Colorectal cancer	Almokadem and Belani, 2012; W. Zhu et al., 2023
α3β1	Blocking binding to laminin Targeting interactions with CD151	Breast cancer Lung cancer	Subbaram and Dipersio, (2011)
α2	Reducing EMT cell migration capability Restoring integrin surveillance system Inhibiting cell adhesion activation affecting the Hippo pathway	Colon cancer Hepatocellular carcinoma	Wong et al., 2016; Ferraro et al., 2013
β7	Inducing conformational changes to expose multiple myeloma (MM)-specific antibody epitopes	Multiple Myeloma	Hosen et al. (2017)

## 4 The role of integrins in stem cells

The self-renewal and proliferation of stem cells are tightly controlled by the synergistic interaction of intrinsic factors and signals within the stem cell niche (Chen, Lewallen, and Xie, 2013). The integrin family, as primary adhesion molecules, is highly expressed in various stem cell niches. They participate in stem cell homing, mediate adhesion to maintain stem cells within the niche, regulate intercellular signals from the niche that modulate stem cell proliferation, and mediate adhesion and asymmetry in stem cell division (Ellis and Tanentzapf, 2010).

The main role of integrins in stem cells is to participate in stem cell maintenance, proliferation, and regulation of stemness functions. Stem cell proliferation primarily occurs through asymmetric cell division. In *Drosophila* intestinal stem cells, integrins have been found to regulate cell polarity by interacting with ECM, controlling the asymmetric distribution of proteins into daughter cells, thus maintaining stem cell proliferation (Anderson, Owens, and Naylor, 2014). Moreover, studies have shown that conditional depletion of ITGB1 in basal mouse mammary stem cells disrupts the asymmetric pattern of cell division necessary for maintaining the microenvironment (Isomursu et al., 2019). In the regulation of stem cell function, research has identified Periostin (Postn) as a regulator of stem cell homing and niche formation by interacting with various integrins. For instance, integrins can act as receptors for Postn, mediating the maintenance of neural stem cell function (Suresh et al., 2019). However, due to low expression of ITGB1 in neural stem cells and limited interaction with ECM rich in laminin, it is believed to contribute to the relative quiescence of neural stem cells (Isomursu et al., 2019).

More importantly, in recent years, an increasing body of research has found that integrins not only promote the maturation and differentiation of luminal cells in the mammary gland, but also mediate the differentiation of bipotent pancreatic progenitor cells into ductal or endocrine lineages (Isomursu et al., 2019). Particularly critical among these is integrin αvβ3. The breast is one of the most active organs in adult females, undergoing hormonal-driven activation of stem cells and remodeling of epithelial cells during menstrual cycles and pregnancy (Fu et al., 2017; Asselin-Labat et al., 2006; Prater et al., 2014). Previous studies have revealed that the cell surface receptor integrin αvβ3 acts as a key switch activated during pregnancy to mobilize stem cells for epithelial remodeling (Desgrosellier et al., 2014). ITGB3 is essential for pregnancy-associated breast development. The β3 cytoplasmic signaling domain promotes P12.5 MaSC clone activation and mammary gland development during pregnancy, but does not affect ductal epithelial cells. TGFβ2 stimulates ITGB3 expression in MaSCs/basal cells and enhances MaSC colony formation in an ITGB3-dependent manner, thereby promoting pregnancy-associated mammary gland development (Desgrosellier et al., 2014).

## 5 The role of integrins in cancer stem cells

### 5.1 Cancer stem cell

Tumor stem cells are a subset of tumor cells possessing stem cell properties. They exhibit self-renewal and self-differentiation capabilities, giving rise to heterogeneous tumor cell populations. These tumor cells lose their self-renewal capacity (Clevers, 2011). In 2003, Al-Hajj et al. first isolated CD44^+^ CD24-/low breast cancer stem cells from breast cancer tissues, providing the initial evidence of the presence of tumor stem cells in solid tumors (Al-Hajj et al., 2003). Tumor stem cells possess potent tumorigenic ability and form the basis for tumor initiation, progression, and maintenance. Moreover, numerous studies have indicated that the presence of tumor stem cells is associated with tumor recurrence, metastasis (Du et al., 2022; Q; Cheng et al., 2022; Primo et al., 2010), and chemotherapy resistance (Guha et al., 2019). To date, tumor stem cells have been found to be closely linked to the occurrence and development of various tumors, including liver cancer, glioblastoma, colorectal cancer, and breast cancer (Ibragimova, Tsyganov, and Litviakov, 2022; Liang et al., 2022; Lathia et al., 2015; Munro et al., 2018).

Various theories exist regarding the origin of tumor stem cells at the current stage. Mainly, “cell fusion,” where a normal stem cell fuses with a tumor cell (S. Zhang et al., 2014; Rizvi et al., 2006), and at the genetic transfer level, DNA from a mutated apoptotic variant cell undergoing division is absorbed by another normal cell or cancer cell (Bjerkvig et al., 2005; Holmgren, Bergsmedh, and Spetz, 2002), as well as mutations occurring during the continuous symmetric division of adult stem cells (Rodriguez, Rubio, and Menendez, 2012; Llaguno et al., 2009), and metabolic reprogramming during somatic or differentiated cell metabolism (Conley et al., 2012), are considered the primary reasons for the generation of tumor stem cells. Additionally, factors such as stress (Fujimori et al., 2012), injury, and ionizing radiation can also induce the production of tumor stem cells. Among these, mutations in normal stem cells are believed to be the main source of tumor stem cells (Passegué et al., 2003).

Due to the similar characteristics shared between tumor stem cells and stem cells, and considering that integrins can facilitate stem cell homing and mediate their maintenance and proliferation, integrins also play a crucial role in regulating tumor stemness.

### 5.2 Integrins can serve as markers for cancer stem cells

**FIGURE 2:**
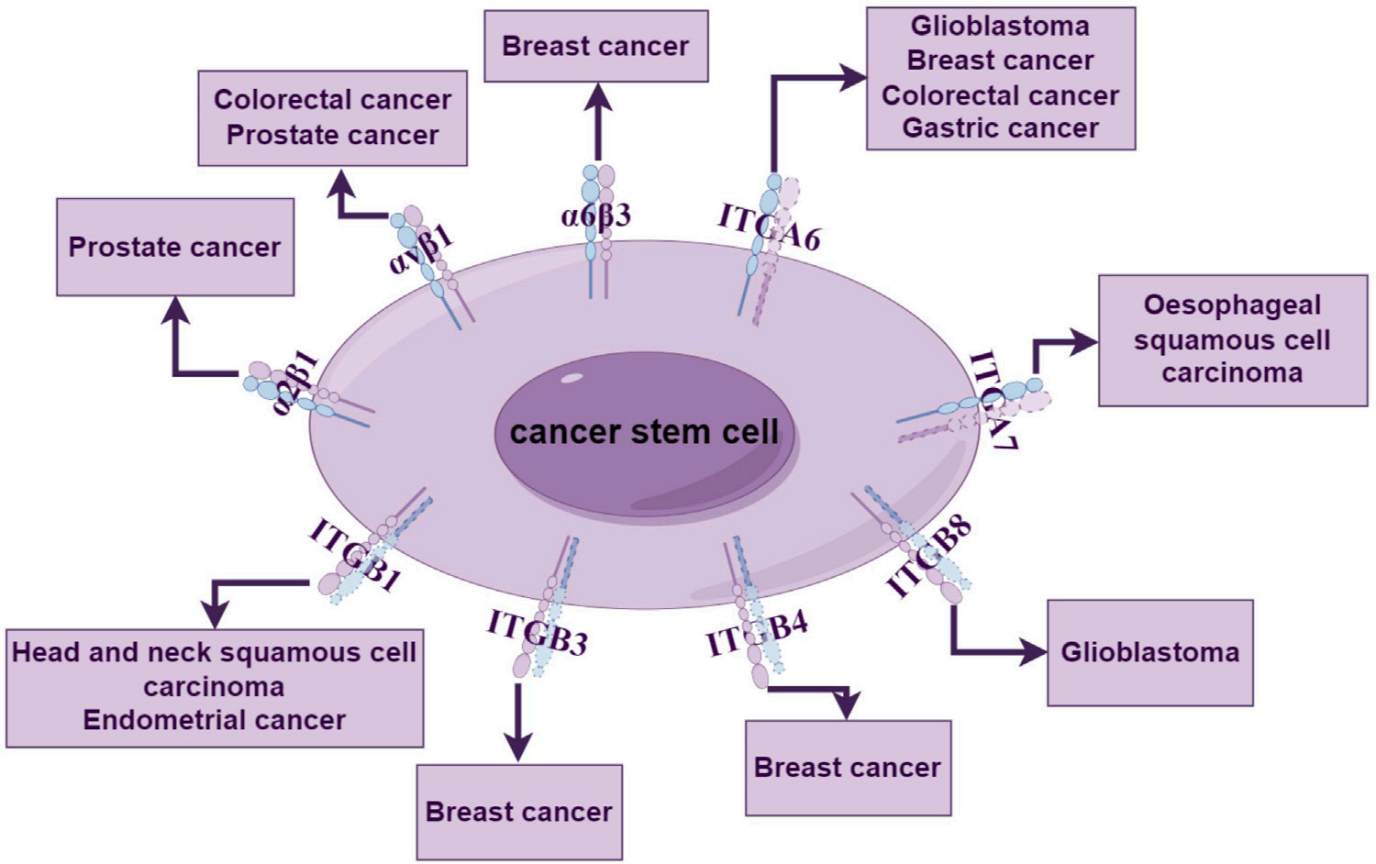
Integrins as tumor stemness markers. Integrin subunits and integrin allosteric dimers can be used as stemness markers for a variety of tumors, including breast cancer, glioma, and prostate cancer, where non-functioning integrin subunits are indicated as dashed lines.

Integrins can influence cellular behavior, including the function and survival of stem cells. Due to the ongoing challenges in isolating tumor stem cells, identifying biomarkers capable of recognizing tumor stem cells is crucial. In existing research, it has been established that most integrins can serve as surface markers for cancer stem cells and can be used to regulate the biological behavior of stem cells and maintain stem cell signaling (Figure 2). When integrin subunits and heterodimers are used as surface markers, they are often named using CD (Cluster of Differentiation).

#### 5.2.1 Integrin α2β1 (CD49b)

Integrin α2β1 was initially identified as an extracellular matrix receptor for collagen and/or laminin. In prostate cancer, the integrin heterodimer α2β1 has been identified as a marker for stemness. Studies have shown that the sorted α2β1^+/hi^ cell subset exhibits higher clonogenic and clonogenic potential *in vitro*, which may be associated with a higher proportion of prostate cancer progenitor cells within the α2β1^+/hi^ cell subset (Patrawala et al., 2007).

#### 5.2.2 Integrin αVβ1 (CD51)

Research has found that CD51^+^ colon cancer stem cells exhibit more pronounced sphere-forming ability and migration and invasion potential *in vitro* compared to CD51^−^cells. *In vivo*, they can increase tumor occurrence and metastasis and enhance tumor chemoresistance, suggesting they serve as a novel functional marker for colon cancer stem cells (Wang J. et al., 2017). Additionally, CD51can also be used to enrich prostate cancer stem cells. The isolated CD51^+^ cell population exhibits higher expression levels of stemness-related genes (Sox2, Nestin, and Nanog), more ALDH + cells, and a stronger ability to form larger colonies *in vitro* (Sui et al., 2018).

#### 5.2.3 Integrin α6 (CD49f, ITGA6)

In breast cancer, CD49f is commonly used in conjunction with other integrin subunits to enrich breast stem cells via FACS. Breast cancer cells co-labeled with CD49f+/CD61+ (ITGB3) exhibit enhanced tumor sphere-forming ability, tumorigenicity, and resistance to chemotherapy drugs (Lo et al., 2012).

ITGA6 has been identified as a stemness marker for various tumor stem cells. In glioblastoma, ITGA6 is more abundant in glioblastoma stem cells (GSCs) compared to non-glioma stem cells. Analysis of tumor spheres has revealed co-expression of ITGA6 with the tumor stem cell markers CD133, nestin, and Olig2. Moreover, ITGA6 can be used to enrich cells with *in vitro* GSC characteristics in tumors with barely detectable levels of CD133 expression (Lathia et al., 2010). Additionally, studies have shown that enriching for CD49f can enhance tumor sphere formation efficiency and Tumor-initiating cells (TIC) activity in metastatic breast cancer models (Brooks et al., 2016). CD49f+ cell populations can effectively enrich colon cancer stem cells, which exhibit higher tumorigenicity and differentiation capacity (Haraguchi et al., 2013). Similarly, in gastric cancer cells, only CD49f^high^ gastric cancer cells can grow and form tumor stem cell spheres with strong tumorigenicity (Fukamachi et al., 2013).

#### 5.2.4 Integrin α7 (ITGA7)

The ITGA7 (+) cells isolated from esophageal squamous cell carcinoma and cells overexpressing ITGA7 exhibit stronger stemness characteristics, including elevated expression of stemness-related genes and enhanced abilities in self-renewal, differentiation, and resistance to chemotherapy. Therefore, ITGA7 represents a potential functional stem cell marker for esophageal squamous cell carcinoma (Ming et al., 2016). Additionally, ITGA7 is expressed in tongue squamous cell carcinoma, where ITGA7+ cells exhibit higher expression of stem cell markers CD44 and CD133 compared to ITGA7-cells (Lv, Yang, and Yang, 2020).

#### 5.2.5 Integrin β1 (ITGB1)

From head and neck squamous cell carcinoma (HNSCC) parent cell lines, ITGB1 (+) cells were sorted, and it was found that in mouse xenograft models, ITGB1 (+) cells exhibited greater tumorigenicity compared to ITGB1 (−) cells. Additionally, HNSCC cells with high expression of ITGB1 showed stronger self-renewal capacity. Therefore, it is suggested that ITGB1 can serve as a marker for the stemness of head and neck squamous cell carcinoma (Moon et al., 2019; Lin et al., 2014). In endometrial cancer, ITGB1 has been identified as a surface marker for enriching endometrial cancer stem cells. The CSC activity and self-renewal capacity of ITGB1 (+) cell populations were found to be higher than those of other primary endometrial carcinoma (EC) cells (Y.H. Huang et al., 2024).

#### 5.2.6 Integrin β3 (CD61, ITGB3)

A study identified a population of cancer stem cells (CSCs) in a mouse model of breast cancer (MMTV-WT-1) using the luminal epithelial progenitor marker ITGB3 (CD61). This population demonstrated a significantly enriched tumorigenic capacity compared to the CD61 (−) subset (Vaillant et al., 2008).

#### 5.2.7 Integrin β4 (CD104, ITGB4)

Breast cancer cells can also utilize surface ITGB4 to isolate a highly enriched population of human breast cancer stem cells (CSCs), and the gene regulatory network operating in ITGB4 (+) CSCs has been identified (Lambert et al., 2022). Furthermore, dividing triple-negative breast cancer cells into ITGB4^hi^ and ITGB4^lo^ subsets revealed that the ITGB4^hi^ subset possesses stronger tumor-initiating capabilities (Bierie et al., 2017). These studies collectively suggest that ITGB4 can serve as a stemness marker for breast cancer.

#### 5.2.8 Integrin β8 (ITGB8)

ITGB8 has also been found to enrich glioblastoma stem cells (GSCs), with co-expression of ITGB8 and the stemness marker SOX2 observed in ITGB8 (+) glioblastoma cells (Malric et al., 2019). Another study identified high expression of ITGB8 in GSCs, which correlated positively with stemness markers. After sorting cells into ITGB8+ and ITGB8- groups and culturing them separately, it was discovered that the ITGB8+ cell population exhibited stronger proliferation and self-renewal capabilities (Y. Liu et al., 2022).

### 5.3 Integrins regulate the tumor stemness of solid tumors

**FIGURE 3:**
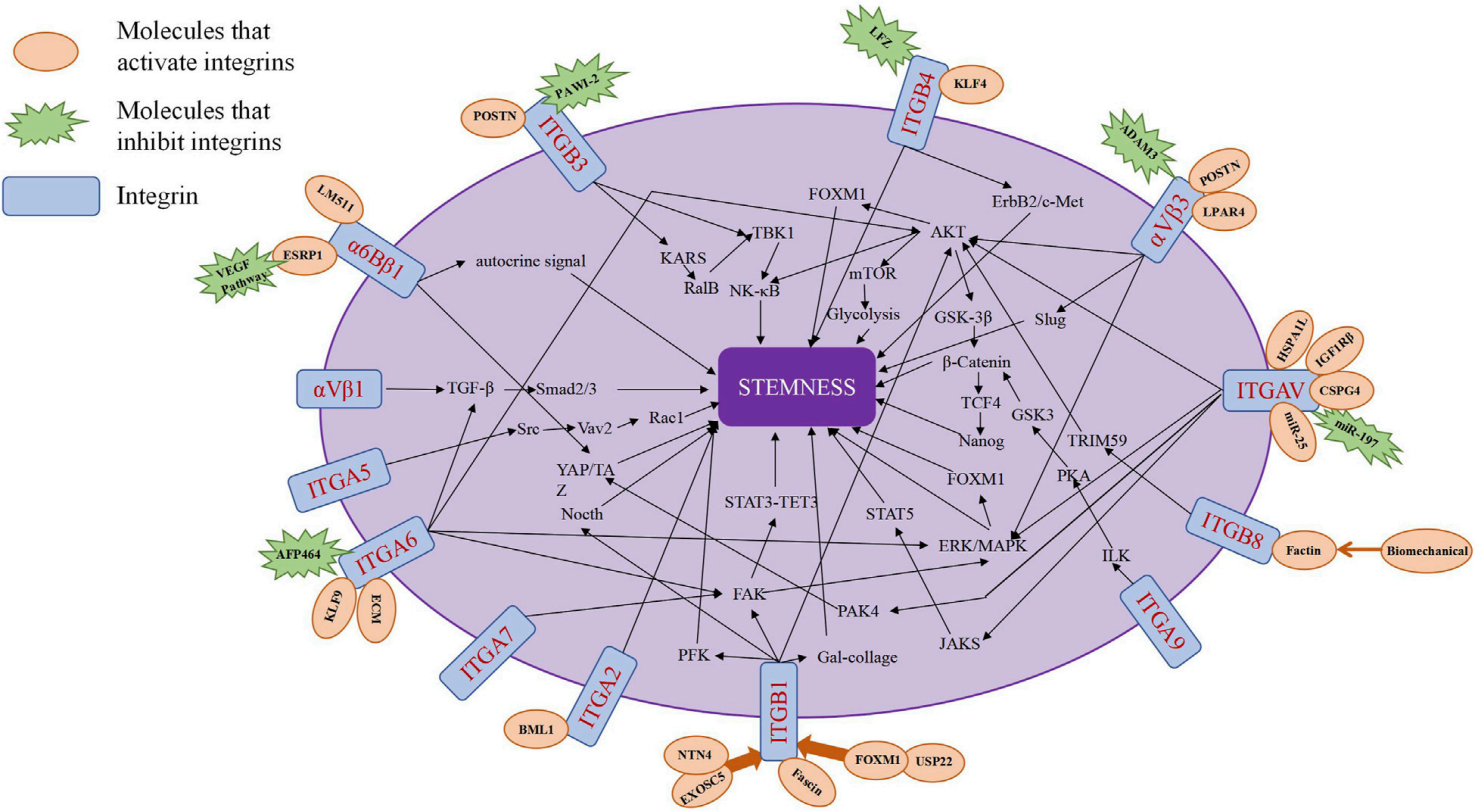
The major pathways of integrin regulation of tumor stemness.

Integrins can serve as stemness markers for identifying tumor stem cells within tumor tissues. Additionally, integrins and integrin signaling pathways can act as regulatory factors for tumor stemness, promoting the growth and proliferation of tumor stem cells (J. Xiong et al., 2021) (Table 2; Figure 3).

**TABLE 2:** Major mechanisms of integrin regulation of stemness in various types of tumors.

Integrin	Mechanism	Tumor	References
αVβ1	TGF-β-Smad2/3	Colorectal Cancer, Pancreatic Cancer	Wang et al. (2017b), B. Zhang, Ye, et al., 2019
αVβ3	αvβ3-Akt/Erk-FOXM1	Pancreatic Cancer	(J. Cao et al., 2019)
	PAK4-YAP/TAZ	Glioblastoma	Cosset et al. (2017)
	αvβ3-slug	Breast Cancer	Desgrosellier et al. (2014)
	POSTN-αvβ3		Lambert et al. (2016)
	ADAM23-αvβ3	Lung Cancer	Ota et al. (2016)
α6Bβ1	Hippo	Breast Cancer	Chang et al. (2015)
	a6Bβ1-autocrine signal		Goel et al. (2013)
α2	ITGA2-BMI1	Glioblastoma	Vora et al. (2019)
αV	ERK/MAPK	Glioblastoma	Niibori-Nambu et al. (2024)
	STAT5	Prostate Cancer, Lung Cancer	Ju et al., 2021; Lee et al., 2019
	ITGAV-miR-25	Prostate Cancer	Zoni et al. (2015)
	AKT/NF-κB, AKT/GSK3β/β-catenin	Pancreatic Cancer	Choi et al. (2020)
α5	Src/Vav2/Rac1	Breast Cancer	Xiao et al. (2018)
α6	ZEB1/YAPI, ERK, FAK	Glioblastoma	Kowalski-Chauvel et al., 2019; Velpula et al., 2012; Herrmann et al., 2020
	KLF9-ITGA6		Ying et al. (2014)
	Integrin-TGFβ	Breast Cancer	Lo et al. (2012)
	AKT	Head and Neck Squamous Cell Carcinoma	An et al. (2021)
α7	FAK-ERK1/2	esophageal squamous cell carcinoma, hepatocellular carcinoma	Zhao et al., 2022; Lv, Yang, and Yang, 2020
α9	ITGA9-β-catenin	Breast Cancer	(Z. Wang et al., 2019)
β1	Fascin-ITGB1	Breast Cancer	(Barnawi et al., 2019)134
	USP22-FOXM1-ITGB1		(K. Liu et al., 2023)
	ITGB1-PFK	Colorectal Cancer	Han et al. (2022)
	Integrin β1/AKT/GSK-3β/β-catenin/TCF4/Nanog	Hepatocellular Carcinoma	(R. Zhang et al., 2017)
	ITGB1-Gal-collagen	Lung Cancer	Gardelli et al. (2021)
	EXOSC5- NTN4/ITGB1	Endometrial Carcinoma	(Y.H. Huang et al., 2024)
	ITGB1-Notch1	Head and Neck Squamous Cell Carcinoma	Moon et al. (2019)
β3	ITGB3-KRAS	Pancreatic Cancer	(J. Cheng and Cashman, 2020)
	POSTN-ITGB3	Breast Cancer	Lambert et al. (2016)
	NF-κB	Melanoma	(X. Zhu et al., 2019)
β4	KLF4-ITGB4	Glioblastoma	Ma et al. (2019)
	ErbB2/c-Met	Prostate Cancer	Yoshioka et al. (2013)
β8	F-actin/ITGB8/TRIM59/AKT/mTOR/Glycolysis	Prostate Cancer	Qiu et al. (2024)

#### 5.3.1 Integrin αvβ1 (CD51/CD29)

Integrin αvβ1 (CD51/CD29) is highly expressed in pancreatic cancer cells. However, the expression of αvβ1 in pancreatic cancer cells does not directly affect cancer cells. Instead, αvβ1 secreted by macrophages promotes tumor initiation and the self-renewal of tumor stem cells in pancreatic cancer through the TGF-β1-Smad2/3 axis (Zhang et al., 2019). Furthermore, CD51 is functionally involved in maintaining the stemness phenotype of colorectal cancer. The CD51^+^ cell population promotes colorectal cancer tumor sphere formation, cell motility, and tumorigenicity by activating the TGF-β pathway (Wang Q. et al., 2017).

#### 5.3.2 Integrin αvβ3

Glioma stem cells (GSCs) not only possess the ability of self-renewal and self-differentiation, but also survive in glucose-deficient environments by upregulating the high-affinity glucose transporter Glut3 (Flavahan et al., 2013). Expression of integrin αvβ3 in patient-derived glioma spheres is considered essential for GLUT3. Research indicates that integrin αvβ3 regulates GLUT3 expression through the PAK4-YAP/TAZ axis, thereby controlling the dependence of patient-derived glioma spheres on GLUT3, offering an effective target for combating the most invasive and drug-resistant cancer stem cells (CSCs) (Cosset et al., 2017). Additionally, CSCs play a significant role in the development of glioma therapy resistance. Studies demonstrate that TR-Lip can safely traverse the blood-brain barrier by binding to integrin αvβ3, destroying glioma cells and transporting glioma stem cells, while disrupting vascular mimicry (Vm) channels, thereby providing a secure drug delivery system for glioma treatment (Shi et al., 2015).

In human breast cancer cells, αvβ3 plays a crucial role in activating adhesion proteins, forming tumor spheroids, and initiating tumorigenesis. Research suggests that tumor-derived Periostin (POSTN) and ITGB3 are key components in maintaining breast cancer stem cells (BCSCs), but there is significant variability in their ability to initiate mammosphere growth, with only MIII cells expressing high surface levels of αvβ3 forming stable mammospheres (Lambert et al., 2016). In pregnancy-associated breast cancer, αvβ3 promotes the stem-like characteristics of breast cancer cells through Slug activation, thereby initiating tumor growth (Desgrosellier et al., 2014). However, αvβ3 exerts specific effects on tumor-initiating cells without affecting the fundamental proliferative and survival responses required for primary tumor growth (Desgrosellier et al., 2014). Additionally, breast cancer stem cells exhibit strong resistance to doxorubicin. In the study by Wang et al., doxorubicin significantly induced expression of integrin αvβ3 and CD90, and CS-V peptide nanoparticles targeting integrin αvβ3 promoted the cytotoxic effect of doxorubicin on breast cancer stem cells (W. Wang et al., 2021). Therefore, research suggests that integrin αvβ3 participates in the positive regulation of tumor stemness and indicates its potential as a target for combating BCSCs. Under stress conditions, upregulation of the G protein-coupled receptor lysophosphatidic acid receptor 4 (LPAR4) drives stress tolerance in breast cancer stem cells (CSCs) and promotes the initiation of cancer stem cells and tumors by activating downstream fibronectin-binding integrins α5β1 and αvβ3 (Wu, Weis, and Cheresh, 2023). Consequently, CSCs possess inherent characteristics that enable them to overcome stressors within the microenvironment, including hypoxia, nutrient deprivation, loss of appropriate ECM, and chemotherapy (T. Huang et al., 2020).

Pancreatic cancer, often dubbed as the “king of cancers” in the digestive system, is influenced by periostin-mediated activation of integrin αvβ3-Akt/Erk-FOXM1 signaling, promoting epithelial-mesenchymal transition (EMT) and stemness features in pancreatic cancer stem cells (CSCs) (J. Cao et al., 2019). Additionally, integrin αvβ3 overexpression has been validated in human pancreatic cancer stem cells, emphasizing its role as a driving force for pancreatic cancer stemness (T. Fang et al., 2022).

Integrin signaling is involved in maintaining stemness in hepatocellular carcinoma (HCC). Studies have shown that osteopontin (OPN), a tumor protein, binds to αvβ3, activating the transcription factor NF-κB, leading to the upregulation of HIF-1α transcription and its downstream target gene BMI1. This process mediates the maintenance of a stem cell-like phenotype and enhances the self-renewal capacity of liver cancer stem cells (L. Cao et al., 2015).

Cancer stem cells in various cancer tissues and cell lines. The presence of integrin αvβ3 has also been observed in lung cancer SP cell subpopulations. Studies indicate that in lung cancer cells, ADAM23 interacts with integrin αvβ3 to exert inhibitory effects on cancer progression, suggesting that downregulation of ADAM23 in SP cells may contribute to enhancing the phenotype of tumor stem cells by promoting the activity of integrin αvβ3 (Ota et al., 2016).

#### 5.3.3 Integrin α2 (ITGA2)

ITGA2 has been identified as a key downstream target of BMI1 in CD133+ glioblastoma stem cells (GBM-SCs), regulating the characteristics of GBM stem cells (Vora et al., 2019).

#### 5.3.4 Integrin αV (ITGAV)

MiR-197 has been shown to regulate the expression of ITGAV via the STAT5 pathway, thereby affecting the proliferation, invasion, and metastasis of prostate cancer cells, while miR-197 can also inhibit the growth of prostate cancer by targeting ITGAV and regulating the development of PCSCs through the STAT5 pathway (Ju et al., 2021). Additionally, researchers have found that the expression of ITGAV and ITGA6 is higher in ALDH^high^ prostate cancer cells compared to ALDH^low^ cells (Zoni et al., 2015). Both ITGAV and ITGA6, as functional targets of miR-25, can regulate the migration and invasion of highly metastatic PCSCs by binding with miR-25 (Zoni et al., 2015). ITGAV, as a functional molecule, serves as a marker of tumor stem cells. Its expression in prostate cancer correlates positively with other stem cell markers such as CD44 and ALDH. In prostate cancer cells with metastatic initiation ability, the expression of ITGAV functionally participates in the acquisition and maintenance of prostate cancer stemness. Moreover, ITGAV is also involved in the formation and maintenance of prostate CSC pools, playing a crucial role in prostate tumor initiation (Levi et al., 2021; Sui et al., 2018).

Furthermore, IL-32γ overexpression can promote the growth and apoptosis of lung cancer stem cells, but this promotive effect can be reversed by ITGAV, indicating that the STAT5 pathway mediated by ITGAV can promote the growth of lung cancer stem cells (Lee et al., 2019). Additionally, ITGAV forms a complex with heat shock 70-kDa protein 1-like (HSPA1L) and IGF1Rβ, participating in the activation of IGF1Rβ. The activation of IGF1Rβ associated with the HSPA1L/ITGAV complex enhances the tumor stemness and radiotherapy resistance of non-small cell lung cancer through downstream AKT/NF-κB or AKT/GSK3β/β-catenin activation pathways (Choi et al., 2020).

Research has shown that ITGAV can interact with CSPG4, regulating the microenvironment of Glioma Initiating Cells (GICs) by upregulating Integrin-ERK/MAPK signaling, thereby promoting the maintenance and differentiation of GICs and leading to tumor formation (Niibori-Nambu et al., 2024).

#### 5.3.5 Integrin α5 (ITGA5)

Similarly, ITGA5 can interact with miR-205, affecting the stemness and metastasis of TNBC via the Src/Vav2/Rac1 pathway. ITGA5 is significantly upregulated in CD44+EpCAM + gastric cancer stem cells (Xiao et al., 2018). Targeting ITGA5 can weaken the tumor sphere formation ability of gastric cancer stem cells and suppress the stem-like characteristics of gastric cancer (Li et al., 2023).

#### 5.3.6 Integrin α6 (ITGA6)

At the molecular level, various cancer-related pathways (such as FAK, ERK/MAPK, Src, AKT, and Ras) are activated due to elevated levels of ITGA6, thereby promoting the stemness of glioblastoma. ITGA6 can regulate the expression of FGFR1 in glioblastoma stem cells through the ZEB1/YAP1 transcription complex and upregulate the proliferation and stemness of GBMSCs by synergistically interacting with FGFR1 (Kowalski-Chauvel et al., 2019). Aberrant ERK signaling promotes cancer invasiveness, and GSCs rely on ERK signaling transduction to regulate the interaction between N-cadherin and ITGA6, thereby modulating GSC invasion *in vitro* (Velpula et al., 2012). Activation of the ITGA6-FAK pathway increases the activity of STAT3 and the expression of TET3 and 5hmC levels in GSCs(Herrmann et al., 2020). The ECM-Integrin α6-STAT3-TET3 axis regulates the hydroxymethylation of key genes in GSCs, thereby increasing the carcinogenicity of GSCs and their resistance to therapy (Herrmann et al., 2020). Additionally, studies suggest that the regulatory role of ITGA6 on glioblastoma stemness may be controlled by Kruppel-like factor-9 (KLF9) (Ying et al., 2014). Researchers have identified three subgroups of stem cells in glioblastoma stem cells: mesenchymal (MES), proneural (PN), and classical (CL) (Wang Q. et al., 2017), with ITGA6 maintaining the stemness of PN-GSCs but enhancing the radiotherapy resistance of MES-GSCs(Stanzani et al., 2021).

Moreover, ITGA6 can promote the growth of tumor-initiating cells (TICs). Studies have found that in breast cancer cells, the AhR agonist Aminoflavone pro-drug (AFP464) can inhibit the formation of breast tumor spheres and suppress TIC growth by inhibiting ITGA6 expression in breast cancer (Brantley et al., 2016). CD61 and CD49f can also identify HER2/neu induced breast tumor-initiating cells and regulate the self-renewal of breast cancer-initiating cells through the Integrin-TGFβ signaling, maintaining the stemness of breast tumors (Lo et al., 2012). ITGA6 has two variants: α6A and α6B, with past studies suggesting that the α6A isoform does not maintain tumor stem cell function (Goel et al., 2014), while breast cancer cells with high expression of integrin α6Bβ1 exhibit CSC characteristics (Chang et al., 2015). Breast cancer stem cells produce a laminin (LM) 511 matrix, which acts as a ligand for integrin α6Bβ1, promoting self-renewal of breast cancer stem cells and breast cancer development by binding to integrin α6Bβ1 and activating the Hippo signaling factor TAZ (Chang et al., 2015). Integrin α6β1 drives TNBC tumor initiation by mediating the activation of autocrine signals (Goel et al., 2013), while the autocrine VEGF signaling pathway maintains the expression of the α6B variant by inhibiting the splicing factor ESRP1, thereby maintaining tumor stem cells and promoting tumor formation (Goel et al., 2014).

Furthermore, in HPV-positive HNSCC cells, ITGA6 can regulate the stemness of tumor cells through the AKT pathway, affecting the size and number of tumor cell spheres formed in HPV-positive HNSCC cells and enhancing the self-renewal capacity of tumor stem cells (An et al., 2021).

#### 5.3.7 Integrin α7 (ITGA7)

Calcium channel α2δ1 subunit-positive hepatocellular carcinoma (HCC) TICs initiate ECM remodeling by secreting LOX, leading to the formation of abundant cross-linked collagen fibers around the tumor (Zhao et al., 2022). This process is driven by the ITGA7-FAK-ERK1/2 signaling pathway, facilitating the acquisition and maintenance of HCC TIC characteristics (Zhao et al., 2022). In esophageal squamous cell carcinoma (OSCC), ITGA7 regulates CSC properties through the activation of FAK-mediated signaling pathways (Ming et al., 2016). Knocking out ITGA7 effectively reduces the stemness of OSCC cells (Ming et al., 2016).

#### 5.3.8 Integrin α9 (ITGA9)

β-catenin is a crucial regulatory protein in the WNT signaling pathway. ITGA9 promotes the stemness, angiogenic ability, and metastatic potential of triple-negative breast cancer (TNBC) by inhibiting the degradation of β-catenin (Z. Wang et al., 2019). Further research indicates that ITGA9’s effect on β-catenin is mediated through the ILK/PKA/GSK3 pathway (Z. Wang et al., 2019).

#### 5.3.9 Integrin β1 (CD29, ITGB1)

Breast cancer is one of the most common and highest incidence tumors among women. Studies suggest the presence of a subpopulation of cells with stem cell-like properties within breast cancer (Rosen and Jordan, 2009), identified using stem cell markers CD44^+^/CD24^-^. USP22 and Fascin regulate the transcription of ITGB1, influencing the self-renewal and metastasis of breast cancer stem cells. USP22, acting as a deubiquitinating enzyme, inhibits the degradation of FOXM1 in breast cancer, thereby upregulating the transcription of ITGB1 and supporting the self-renewal of breast cancer stem cells (K. Liu et al., 2023). *In vitro* studies show that Fascin directly affects the expression of ITGB1 in breast cancer, thereby affecting the self-renewal capacity of breast cancer cells and their resistance to chemotherapy (Barnawi et al., 2019).

Integrin signaling can promote the dedifferentiation of colorectal cancer cells, enabling tumor cells to acquire a stem cell-like phenotype with self-renewal, differentiation, and tumorigenic abilities. For example, 3D-cultured colorectal cancer cells can promote tumor dedifferentiation through an ITGB1/PFK-dependent mechanism (Han et al., 2022). POSTN regulates the stemness of HCC cells by activating the integrin β1/AKT/GSK-3β/β-catenin/TCF4/Nanog signaling pathway (R. Zhang et al., 2017).

High expression of ITGB1 has been found in lung cancer stem cells, and studies indicate that the binding of ITGB1 to collagen promotes the expansion and generation of CSCs, thereby enriching tumor stem cells in lung cancer (Gardelli et al., 2021). Similarly, in oral tumors, ITGB1 expression is increased in oral cancer stem cells, and upregulation of ITGB1 in oral cancer stem cells leads to increased expression of other stemness markers and an increase in the size of stem cell spheres (Park et al., 2023; Lin et al., 2014).

Endometrial cancer is one of the most common tumors in the female reproductive system. Studies show that ITGB1 is used to enrich endometrial cancer stem cells, and EXOSC5 regulates the signaling of ITGB1 through the NTN4/ITGB1 axis, enhancing the cancer stemness of endometrial cancer stem cells (Y.H. Huang et al., 2024). Additionally, ITGB1 interacts with Notch1 to regulate the stemness of head and neck squamous cell carcinoma, thereby controlling the self-renewal, differentiation, and *in vivo* tumorigenicity of cancer cells (Ming et al., 2016).

#### 5.3.10 Integrin β3 (CD61, ITGB3)

The regulatory role of ITGB3 in tumor stemness has been identified in breast cancer, pancreatic cancer and melanoma. The complete POSTN-ITGB3 signaling axis is essential for maintaining breast cancer stem cells (CSCs). Research indicates that loss of POSTN or ITGB3 can attenuate NF-κB transcriptional activity (Lambert et al., 2016). The POSTN-ITGB3 signaling axis regulates cytokine production, thereby activating the STAT3 signaling pathway. By controlling cytokine production, it alters the cytokine-STAT3 balance, which is advantageous for breast CSCs(Lambert et al., 2016).

In a highly validated CSC model, PAWI-2 inhibits Integrin β3-KRAS signaling, thereby suppressing self-renewal capacity and cell viability of pancreatic cancer CSCs (J. Cheng and Cashman, 2020). On the surface of melanoma cancer cells, ITGB3 forms a membrane-proximal complex with KRAS and Galectin-3, recruiting RalB and leading to TBK1 activation. Phosphorylated TBK1 promotes activation of the NF-κB signaling pathway, promoting a stem-like phenotype in melanoma and serving as a driver of chemoresistance in melanoma therapy (X. Zhu et al., 2019).

#### 5.3.11 Integrin β4 (CD104, ITGB4)

Glioblastoma (GBM) is one of the most common malignant tumors in the intracranial region. Targeting GSCs may become a primary approach for GBM treatment. Researchers have found that ITGB4 is highly expressed in GSCs and is involved in a feedback loop with Kruppel-like factor-4 (KLF4). The mutual regulation between KLF4 and ITGB4 promotes the self-renewal, proliferation, and migration of GSCs (Ma et al., 2019).

Prostate cancer has an extremely high incidence among genitourinary tumors. Studies have found high levels of expression of ITGB4 in prostate cancer, where ITGB4 in prostate tumor progenitor cells amplifies ErbB2 and Met signaling transduction. Through the regulation of ErbB2 and c-Met signals, ITGB4 promotes the self-renewal and transportation amplification of prostate tumor progenitor cells, as well as rapid proliferation of tumor cells (Yoshioka et al., 2013).

Additionally, resistance in lung cancer is also associated with the presence of cancer stem cells (CSCs). Research indicates that carfilzomib (CFZ) acts synergistically with cisplatin to inhibit CSC growth by suppressing the expression of ITGB4 and SOX2. Therefore, targeting cancer stem cells plays a crucial role in inhibiting lung cancer resistance (Guo et al., 2023).

#### 5.3.12 Integrin β8 (ITGB8)

In bladder cancer, it is believed that tumor cells with inherent plasticity possess the functionality of cancer stem cells. Biomechanical stimulation generated by three-dimensional Matrigel activates the F-actin/ITGB8/TRIM59/AKT/mTOR/glycolysis pathway, thereby enhancing the flexibility and tumorigenicity of tumor cells (Qiu et al., 2024).

## 6 Summary and outlook

Integrins, as cell adhesive molecule, play crucial roles in cancer development, participating in various aspects including initiation, growth, metastasis, invasion, and drug resistance. Tumor stem cells are the primary cause of tumor metastasis, invasion, recurrence, and drug resistance, making them the key focus of tumor therapy in recent years. In the regulation of cancer stem cells (CSCs), integrins modulate the functions and characteristics of CSCs through multiple pathways, making them pivotal factors in the regulation of tumor stemness. Consequently, CSCs emerge as effective therapeutic targets, and inhibition of CSC self-renewal and differentiation holds promise for effectively restraining tumor progression *in vivo*. Future research will continue to delve into the mechanisms underlying integrin regulation of CSCs and develop therapeutic strategies targeting CSCs, paving the way for breakthroughs in cancer treatment.

Currently, most studies suggest that large-scale tumor recurrence relies on tumor stem cells with self-renewal and differentiation capabilities. Tumor stem cells remain in a quiescent state (G0 phase) of cellular differentiation and possess robust DNA repair mechanisms and drug exclusion capabilities, rendering traditional treatment regimens incapable of completely eradicating them within tumors. Additionally, factors secreted by tumor stem cells can alter the microenvironment to favor the survival and spread of tumor cells. Therefore, therapeutic strategies targeting tumor stem cells may represent a crucial direction for improving cancer treatment efficacy and reducing tumor recurrence in the future.

Integrins regulate multiple aspects of tumor stem cell characteristics by modulating the composition and structure of the extracellular matrix, controlling interactions between cells, activating multiple signaling pathways, and influencing cell polarity and differentiation status. Targeting integrins as a treatment strategy can effectively impact tumor stem cells through various pathways, including inhibiting homing and colonization, regulating self-renewal and proliferation, reducing metastatic potential, and increasing sensitivity to treatment. Intervention in these mechanisms can help disrupt critical biological features of tumor stem cells, thereby enhancing treatment outcomes and reducing tumor recurrence and metastasis.
